# Empowerment or Threat: Perceptions of Childhood Sexual Abuse in the #MeToo Era

**DOI:** 10.1177/0886260520925781

**Published:** 2020-06-06

**Authors:** Melissa S. de Roos, Daniel N. Jones

**Affiliations:** 1 University of Roehampton, London, UK; 2 University of Nevada, Reno, USA

**Keywords:** sexual abuse, child abuse, reporting/disclosure, sexual assault, cultural contexts

## Abstract

The rise of the #MeToo movement has shed light on the prominence of sexual violence, and its victims who often remain silent. Despite increasing awareness, victims or survivors of sexual violence who disclose may be faced with negative reactions such as disbelief or blame. Such reactions extend to child victims of sexual abuse. This study aimed to shed light on gender differences in responses to sexual violence against a backdrop of #MeToo. Through an online survey (*N* = 253) on Amazon’s Mechanical Turk, we assessed participants’ exposure to and perception of the #MeToo movement. In addition, we measured proximity to a victim or perpetrator of sexual violence. The effect of these variables on participants’ response to a disclosure of childhood sexual abuse was examined. Results indicated that men are more likely to perceive the movement as threatening than women. Furthermore, a discrepancy in proximity to sexual violence emerged, with women more likely to know a victim and men more likely to know a perpetrator. In response to a disclosure of childhood sexual abuse, men were more likely to respond in a skeptical manner than women. Positive perceptions of the #MeToo movement translated into more supportive responses to a disclosure. Proximity to a victim of sexual violence did not impact how people responded to a disclosure, but proximity to a perpetrator was associated with a more negative response. Although the aim of this movement is to give a voice to victims of sexual violence, it may trigger a defensive response from men, which makes them more skeptical toward disclosures of victimization.

The #MeToo movement gained widespread attention on Twitter in October 2017. It was an attempt to highlight the prevalence of sexual violence and to give a voice to victims particularly in the workplace, many of whom had stayed silent before (Smartt, 2017). Ten years before then, counselor Tarana Burke started this movement to draw attention to sexual violence, especially as experienced by members of racial and ethnic minority groups (Snyder & Lopez, 2017).

The hashtag was used as a way to empower female victims of sexual violence and to highlight the prevalence of sexual victimization (Ohlheiser, 2017). In the first 24 hours, 4.7 million people shared the hashtag on Facebook, and 45% of Facebook users had at least one friend who shared the hashtag (Santiago & Criss, 2017). The hashtag trended on Twitter in at least 85 countries (Strum, 2017), showing the international impact of the movement.

Since the rise of the #MeToo movement, responses to the movement have not been universally positive. Criticisms of #MeToo take several forms. For example, people have questioned what its ultimate purpose is (Wilhelm, 2017) and whether it accomplishes any goal. Furthermore, some people have characterized the movement as a witch hunt, and some think that the focus should be only on the worst types of abuse, rather than on mere misconduct, to prevent the public from becoming desensitized (Stephens, 2017). Some have criticized the movement for putting the burden of having to come forward on the victims, with a risk of retraumatizing them in the process (Gerson, 2017). Finally, concerns have been expressed regarding the possibility of false allegations and blaming accused perpetrators without due process (e.g., Cromwell, 2017; Stephens, 2017).

Although it has been suggested that victims should be referred to as survivors, there is some disagreement over the appropriate term (Schwark & Bohner, 2019). It should be the individual’s choice whether they want to be referred to as a victim or survivor. Both terms are imperfect, but “survivor” suggests recovery (Thompson, 2000), which is a complex, ongoing process that the authors cannot assume pertains to all individuals who participated in research. For this reason, we have chosen to use the term “victim”.

## Gender Differences in Responses to Sexual Violence

There is a well-documented gender difference in how people respond to sexual violence, with men more likely to respond skeptically to a disclosure, and to blame the person who makes the disclosure (for a review, see Suarez & Gadalla, 2010). Several explanations for this gender difference have been proposed, and this article will focus on defensive attribution theory (DAT; Shaver, 1970) and proximity to sexual victimization. DAT might be particularly relevant because the high visibility of the #MeToo movement may increase the likelihood of defensive responses.

Research has shown that when people feel threatened because of their membership of a social category, they feel more strongly connected to that category, especially if it represents a minority in society (e.g., Brown & Ross, 1982; Grant, 1992). Social categories people readily identify with include ethnicity and gender (Tajfel & Turner, 1979). Although women have traditionally represented a minority group, initiatives such as the #MeToo movement may operate in a manner that makes women feel empowered in a way they have not felt before. Based on research on social identity, we may expect that because of this shift, some men will feel a stronger identification with other men. This identification may include those accused of sexual violence, when they are confronted with accounts or repostings of #MeToo. The increased attention on mostly women’s experiences of sexual victimization may serve as a threat that triggers some male observers to make defensive attributions in response to disclosures. These defensive attributions (e.g., Walster, 1966) may stem from the perception that they may become the victim of an erroneous claim of causing sexual trauma, which could potentially create perceptions of persecution (Lyon et al., 1994).

Another proposed explanation for gender differences is proximity to sexual victimization, whether through own experiences or those of close others. Several studies have found that people who experienced (sexual) victimization were more likely to believe allegations of sexual violence (Cromer & Freyd, 2007; Miller & Cromer, 2015; Nuttall & Jackson, 1994). These findings fit with the DAT to explain gender differences and expand on it. Women tended to have closer proximity to traumatic events than men, either through their own experiences or the experiences of others close to them (Miller & Cromer, 2015). After controlling for this proximity, gender differences were no longer significant. This finding poses the question with regard to the #MeToo movement: Does the raised awareness increase people’s proximity to sexual violence thereby making them more supportive of disclosures from victims?

On the other side of proximity to victimization, proximity to perpetration may play a role in shaping responses to sexual violence. Antisocial peers are a well-established risk factor for criminal behavior (Gendreau et al., 1992; Humphrey & Kahn, 2000). The chance of engaging in a behavior increases if the individual and their peers have positive views of the behavior (Bagozzi & Burnkrant, 1979). Thus, the importance of association with sexual perpetrators may then play a vital role in how people respond to sexual violence.

Defensive attributions are not limited to victims but may extend to perpetrators. Indeed, if the observer perceives themselves as similar to the perpetrator of a crime, the observer expresses more lenient attitudes toward the perpetrator (Feather, 1996). Back and Lips (1998) hypothesized that the reliable gender differences found in victim-blaming research are a reflection of DAT, with men more likely to perceive similarities between themselves and the usually male perpetrator. More recent studies have found a similar effect when assessing whether perceived similarity indeed affects attributions of blame (e.g., Kahn et al., 2011). These studies found that male observers attribute more blame to female victims than female observers because they do not identify with the female victim to the same extent that female observers do (Davies & Rogers, 2009; Donovan, 2007; Kelly, 2009).

## Childhood Sexual Abuse (CSA) Disclosures

The #MeToo movement has been accused of having too narrow a focus, highlighting victims who “fit” a certain narrative, while excluding, for example, nonheterosexual victims, people of color, and people who are viewed as “undeserving” of empathy (Gill & Orgad, 2018). Another group of victims who may be overlooked is a group who may be at an even greater disadvantage of speaking up and being heard: child victims. It is estimated that one in eight people worldwide are victims of sexual abuse during their childhood (Stoltenborgh et al., 2011). The majority of CSA victims do not disclose the abuse in childhood. Several studies have found that disclosure during childhood is rare, with most victims not disclosing until several years after the abuse took place (Alaggia, 2010; McElvaney et al., 2012).

Unfortunately, even if children do disclose, there is no guarantee that the abuse will stop, as evidenced by high-profile cases such as the Jimmy Savile (“Savile and Hall: BBC ‘missed chances to stop attacks,’” 2016) sexual abuse scandal, and the Larry Nassar case (Chowdhury, 2018). Despite victims disclosing to adults, the abuse was allowed to continue for decades (Bilefsky, 2016; Lavigne & Noren, 2018). These experiences are not exceptions. A recent study showed that victims who came forward with experiences of sexual abuse were only believed in one third of cases, and, in almost half of these cases, no steps were taken to stop the abuse (Stiller & Hellmann, 2017).

## The Present Study

The aim of the present research was to explore people’s exposure to sexual violence and how this relates to their perceptions of sexual violence as well as the #MeToo movement. A recent study explored perceptions of #MeToo and found that men had more negative views of the movement than women (Kunst et al., 2019). The results of this study indicated that underlying ideologies such as sexism shaped people’s perceptions, and that proximity to sexual violence had a comparatively small influence. However, this study did not ask participants about their responses to a specific disclosure scenario, nor did it include proximity to perpetration other than participants’ own perpetration. Case vignettes are the preferred way to assess responses to sexual violence (Font, 2013). Whereas self-report questionnaires have been criticized for pertaining to abstract, artificial, or generalized situations (Alexander & Becker, 1978), vignettes allow for more natural responses to realistic scenarios.

The key question in the present study is whether increased awareness of sexual violence is perceived as a threat, and/or whether it is able to increase perceived proximity to sexual victimization, which may make people more supportive. This study explored exposure to and perceptions of the #MeToo movement and examined the role that proximity to victimization or perpetration plays in shaping responses to a CSA disclosure. Furthermore, with regard to perceptions of the #MeToo movement, we selected the response categories from news articles as well as social media, where the #MeToo movement has been characterized as both positive and negative. Although the victim depicted in the scenario was a child when the abuse happened, at the time of disclosure she is an adult. Given that this is the time when most victims will disclose the abuse for the first time, we feel that this is an important first step in examining how people respond to such a disclosure. In the future, we would like to extend this to vignettes where children disclose sexual abuse. We expected that most participants would have had some exposure to the #MeToo movement. Furthermore, we expected that participants with a proximity to victimization would be more supportive of the movement and a disclosure. In contrast, we predicted that participants with a proximity to perpetration would be more skeptical of the movement and a disclosure. Finally, we expected that gender differences in response to sexual violence could be triggered by a video on #MeToo, with male participants responding more negatively than female participants.

## Method

### Participants

Participant recruitment occurred through Amazon’s Mechanical Turk. Use of MTurk in this study was preferential to college students because college students may represent a well-adjusted, homogeneous population that is not representative of the general population (Buhrmester et al., 2011). Furthermore, data collected on MTurk is found to be equally reliable as data collected through student participant pools (Buhrmester et al., 2011), with some studies suggesting that MTurk participants may be more attentive than more traditional samples (e.g., Hauser & Schwarz, 2016).

A power analysis indicated that 200 participants were required to detect the desired effect size (*R*-squared = .12). To account for missing data and participants who may fail attention checks, 275 participants were recruited for this study. Participants were compensated US$0.80 for their participation. The sample was restricted to participants from the United States, with an approval rate of their work on MTurk of at least 95%, and with at least 50 approved answer submissions.

Any participant who did not stay on the page displaying the video for at least the duration of that video was excluded, as was any participant who could not correctly answer the questions about the video they watched. A total of 253 participants were included in the final sample. The mean age was 35.44 years (*SD* = 11.32), with an age range from 18 to 75 years. There were slightly more male participants (54.2%) and the majority of participants identified as straight (88.5%), bisexual (9.5%), or homosexual (1.2%) (See Supplemental Material online for detailed demographics).

### Materials

#### #MeToo questionnaire

To assess exposure to, and perceptions of, the #MeToo movement, we developed a brief questionnaire. The first four questions ask about exposure to the movement. Two of these ask about proximity to sexual violence either through knowing someone who perpetrated sexual violence or who was victimized. The Inclusion of Other in the Self Scale (IOSS; Aron et al., 1992) is included to assess the degree of perceived closeness. This scale displays seven images of increasingly overlapping circles, representing the participant and someone the participant knows. The participant is then asked to pick the set of circles that best represents their relationship with this person.

The subsequent questions ask about perceptions of and feelings toward the movement. These questions were based upon the criticisms of the movement, as well as on its proposed objectives, to determine the extent to which participants agree with different characterizations of the movement that have been proposed by news and social media. Perceptions include six items (three positive and three negative) that are scored on a 1 to 5 Likert-type scale, ranging from 1 (*strongly disagree*) to 5 (*strongly agree*). Feelings include six feelings (three positive and three negative) that are scored on a 1 to 5 Likert-type scale, ranging from 1 (*strongly disagree*) to 5 (s*trongly agree*). The feelings included emotions that might trigger defensive responses, such as feeling attacked or persecuted.

#### #MeToo manipulation

A 5-min video from *Time Magazine* about the Silence Breakers and the #MeToo movement were used as the manipulation for the #MeToo condition (*Time*, 2017). It includes various accounts, from men and women of different ethnicities and with different backgrounds. It discusses the importance of giving a voice to victims and it encourages viewers to stand up against sexual violence. For the control condition, a 6-min video about pollution in the ocean was watched (*National Geographic*, 2016).

#### CSA vignettes

When using vignettes, it is vital to avoid language that creates demand effects in participants. Examples highlighted by Cromer and Goldsmith (2010) include words like “assault” or “rape” that prime people to think about violence. On the contrary, words like “relationship” or “affair” paint a picture of a consensual relationship. The key then is to focus on neutral, factual language with behavioral descriptors. For this study, the following 125-word vignette was used:

You’re talking with your friend Zoe about #MeToo. She was one of the people who reposted the status on her Facebook wall. She tells you she reposted the status because from when she was thirteen until she was fifteen, her mom’s boyfriend would come into her bedroom at night when everyone was asleep. She tells you he would undress and get under the covers with her. He would touch her and rub against her and “do some other stuff.” It only stopped when her mom broke up with him because she had to move to a different city for her job. Zoe has never told anyone about what happened to her, but now that more people are speaking up, she feels that she can too.

After the vignette, participants were asked the extent to which they believed the CSA account (rate on scale of 1 to 100), how harmful they thought this experience was (1 = *not at all harmful*, 5 = *very harmful*), how much Zoe, Zoe’s mom, and Zoe’s mom’s boyfriend are to blame (rate on scale of 0–100), and how avoidable the experience was (0 = *not at all avoidable*, 5 = *completely avoidable*). These responses to disclosure variables were based on barriers to disclosure as identified by victims. Such barriers include a fear of not being believed or of being blamed, as well as their experience being minimized (e.g., Alaggia, 2010).

#### Positive and Negative Affect Schedule (PANAS)

The PANAS–Short Form (PANAS-SF; Thompson, 2007) is a brief assessment of positive and negative emotions. It uses five items to measure positive emotions and five items to measure negative emotions. Each item is rated on a 5-point Likert-type scale, ranging from 1 (*very slightly or not at all*) to 5 (*extremely*). It was used to assess participants’ feelings after watching one of the videos. The internal reliability in this sample was good (α = .84)

### Procedure

Participants were randomly assigned to the #MeToo manipulation condition or the control condition. After watching the video, participants answered three questions to ensure they paid attention to the video. On the next screen, participants were presented with the vignette. They were asked to carefully read the vignette before moving onto the next screen. The following screens asked the participants to what extent they believed the vignette, how harmful they thought the experience was, who was to blame, and how avoidable they thought the experience described in the vignette was. On the final screen, participants were asked to complete the #MeToo questionnaire.

## Results

### What Are Participants’ Exposure to, and Perceptions of, the #MeToo Movement?

#### Exposure

The majority of participants (91.3%) had heard of the #MeToo movement. The 22 participants who reported they had not heard of the movement were excluded from all analyses that included questions about #MeToo. Nearly 17% of participants stated they had reposted #MeToo. Two out of three participants knew someone who had reposted #MeToo, and nearly 43% of participants knew someone who had done something that would make someone else repost #MeToo.

Next, the closeness to someone who reposted was assessed using the IOSS. A series of seven images depict two circles that increase in overlap, and participants are asked to choose the image that best represents their closeness with the other person. These images were recoded as a 1 to 7 scale, ranging from 1 (*no overlap*) to 7 (*complete overlap*). Participants reported a mean closeness to someone who reposted #MeToo of 3.48 (*SD* = 1.84). The same question was asked for closeness to someone who did something that would make someone else repost #MeToo. A slightly lower mean closeness was reported (*M* = 2.72, *SD* = 1.94).

Independent sample *t* tests were conducted to assess the difference in exposure to the #MeToo movement between men and women. Details are displayed in Table 1

**Table 1.** Descriptive Statistics and Independent Sample *t* Tests of #MeToo Exposure Variables.

Exposure	Men	Women	*t*
	Frequency (%)	Frequency (%)	
Heard of #MeToo	124 (90.5%)	107 (92.2%)	−0.49
Reposted #MeToo	22 (16.1%)	20 (17.2%)	−1.25
Prefer not to say	3 (2.2%)	6 (5.2%)	
Do you know anyone who reposted #MeToo	84 (61.3%)	87 (75%)	−2.33*
Do you know anyone who did something to make someone else repost #MeToo	57 (41.7%)	51 (44.0%)	−0.39

	*M*	*SD*	*M*	*SD*	*t*
Closeness to person you know who reposted #MeToo	3.48	1.94	3.48	1.74	.01
Closeness to person you know who did something to make someone else repost #MeToo	2.94	2.08	2.46	1.74	1.69^†^

† *p* < .10. *p < .05.

. Women were significantly more likely than men to know someone who had reposted #MeToo. Furthermore, the difference in closeness to someone who had done something that would cause someone else to repost #MeToo was marginally significant (*p* = .09), with men reporting a greater closeness than did women.

Finally, correlations were calculated between the exposure variables for men and women.

Details are displayed in Table 2

**Table 2.** Pearson Correlations Between Exposure to #MeToo Variables for Men and Women.

Perceptions	1	2	3	4	5
1. Reposted #MeToo	–	.29***	.45***	.25**	.49***
2. Knowing someone who reposted #MeToo	.25*	–	.33**	.24**	.32**
3. Closeness to someone who reposted #MeToo	.26*	.19***	–	.24*	.73***
4. Knowing someone who did something to make someone else repost #MeToo	.22*	.33**	.27**	–	.28**
5. Closeness to someone who did something to make someone else repost #MeToo	.35**	.27**	.39***	.30**	–

*p < .05. **p < .01. ***p < .001. Men above diagonal.

. For both men and women, all exposure variables were significantly, positively correlated.

#### Perceptions

Perceptions of #MeToo were assessed by asking participants the extent to which the movement could be described by the first six variables displayed in Table 3

**Table 3.** Descriptive Statistics and Independent Sample *t* Tests of Perceptions of #MeToo for Men and Women.

Perceptions	Men		Women		*t*
	*M*	*SD*	*M*	*SD*	
#MeToo is					
Helpful	3.89	1.00	4.24	.88	−2.84**
Important	4.07	1.04	4.50	.78	−3.53**
Divisive	3.15	1.29	2.99	1.40	0.92
Witch hunt	2.68	1.37	2.06	1.32	3.51**
Empowering	3.90	1.02	4.30	.91	−3.9**
Gone too far	2.67	1.38	2.21	1.30	2.56*
#MeToo makes me feel					
Supported	3.15	1.19	3.94	1.00	−5.51***
Persecuted	2.44	1.32	1.78	1.14	4.07***
Worried	2.42	1.31	2.08	1.30	1.96^†^
Empowered	3.08	1.25	4.02	1.13	−5.92***
Heard	3.09	1.30	3.96	1.10	−5.44***
Attacked	2.32	1.38	1.70	1.07	3.86***

† *p* < . 10. *p < .05. **p < .01. ***p < .001.

and to what extent the movement made them feel the last six variables in Table 3. Each answer was scored on a 5-point Likert-type scale, ranging from 1 (*disagree strongly*) to 5 (*agree strongly*). We conducted independent sample *t* tests to compare men and women in their perceptions of the #MeToo movement. The results are displayed in Table 4

**Table 4.** Factor Loadings Based on Principal Component Analysis for Perceptions of #MeToo.

Perceptions	Component	
	1	2
Helpful	.701	
Important	.579	−.315
Divisive		.576
Witch hunt	−.279	.705
Empower	.682	
Gone too far	−.363	.672
Supported	.910	
Persecuted		.904
Worried		.795
Empowered	.918	
Heard	.903	
Attacked		.912

. Every variable showed a significant difference between men and women except divisive and worried.

The perception variables are highly correlated with each other (significant correlations .19–.84; nonsignificant correlations: feeling heard with feeling attacked and feeling heard with feeling worried). Negative perceptions show a strong positive correlation with each other, strong negative correlations with positive perceptions, and vice versa.

Due to these high correlations, a principal component analysis with varimax rotation was conducted to assess the uniqueness of the perception variables. A Varimax rotation was chosen to allow for the possibility that positive and negative perceptions were uncorrelated. Two components with eigenvalues above 1 emerged, and these components explained 68.11% of the variance. The component matrix is displayed in Table 4. Loadings below .25 are not displayed.

The identified factors appear to be positive perceptions and negative perceptions. We created a composite score for positive perceptions by summing the items that indicated positive perceptions of the movement (α = .90). Similarly, a composite score was created for negative perceptions by summing the items indicative of negative perceptions (α = .89). We used these composite scores in all subsequent analyses where perceptions of #MeToo are mentioned. We ran independent sample *t* tests to examine gender differences on positive and negative perceptions. Women (*M* = 25.00, *SD* = 4.97) were significantly more likely than men (*M* = 21.15, *SD* = 5.22) to hold positive perceptions of the movement (*t* = –5.68, *p* < .001). Similarly, men (*M* = 15.70, *SD* = 6.50) were significantly more likely than women (*M* = 12.82, *SD* = 5.91) to hold negative perceptions of the movement (*t* = 3.49, *p* = .001).

#### Vignette variables

Participants were asked seven questions about the vignette describing the CSA disclosure.

Correlations between variables are displayed in Table 5

**Table 5.** Pearson Correlations Between Response to Disclosure Variables for Men and Women.

Response to Disclosure	1	2	3	4	5	6
Believe	–	−.34***	.54***	−.08	.42***	−.13
Blame Zoe	−.32***	–	−.49***	.26**	−.41***	.16
Blame mom’s boyfriend	.33***	−.64***	–	.11	.61***	.03
Blame Zoe’s mom	−.23*	.28**	−.09	–	.08	.19*
Harmful	.38***	−.41***	.63***	−.04	–	−.06
Avoidable	.01	.09	<.01	.35***	.07	–

*p < .05. **p < .01. ***p < .001. Men above the diagonal.

.

Finally, we examined the link between perceptions of #MeToo and responses to the vignette. Positive perceptions of #MeToo were associated with more positive responses than negative perceptions (Table 6

**Table 6.** Correlations Between Perceptions of #MeToo and Responses to a CSA Vignette.

Variable	Positive Perceptions	Negative Perceptions
Believe	.39***	−.38***
Blame Zoe	−.14*	.56***
Blame boyfriend	.15*^†^	−.32**
Blame mom	−.21**	.33***
Harmful	.26***	−.33***
Avoid	−.16*	.19**

*Note*. CSA = childhood sexual abuse.

† *p* < .1. *p < .05. **p < .01. ***p < .001.

). Interestingly, participants with more negative perceptions were more likely to blame Zoe’s mom, and to find the vignette more avoidable.

### Does Proximity to Victimization or Perpetration Affect How Participants Respond to Sexual Violence?

#### Proximity to victimization

First, we computed correlations between proximity to victimization and perceptions of the movement. For men, having reposted #MeToo was associated with both stronger positive (*r* = .19, *p* = .04) and negative (*r* = .38, *p* < .001) perceptions, whereas for women, reposting was only associated with negative perceptions (*r* = .27, *p* < .01). Knowing someone who reposted the status and closeness to such a person was linked to positive perceptions of the movement for both men (*r* = .36, *p* < .001) and women (*r* = .32, *p* = .001).

Second, we performed analyses to assess the effect of proximity to victimization on responses to a CSA disclosure. A series of stepwise multiple linear regressions were conducted for believing the vignette, harmfulness of the vignette, avoidability of the vignette, and amount of blame allocated to Zoe, Zoe’s mom, or Zoe’s mom’s boyfriend. For each regression, the entered variables were knowing someone who reposted the status, closeness to that person, as well as positive and negative perceptions of the movement and gender. Due to skewness of the blame variables, normality assumptions were violated and, as such, a robust estimator (MLR) was used. No significant equations with the proximity to victimization variables emerged.

#### Proximity to perpetration

Correlational analyses showed that men who knew someone who had done something that would make someone else repost #MeToo were more likely to have negative perceptions of the movement (*r* = .25, *p* = .006). Following this, a similar approach, as for proximity to victimization, was taken for the effect of proximity to perpetration. We performed stepwise multiple regression analyses to assess the effect of proximity to perpetration on responses to a CSA disclosure. The variables entered were knowing someone who had done something that would make someone else repost, closeness to this person, positive and negative perceptions toward the movement, and gender. Two significant equations with proximity to perpetration variables emerged. Believing the disclosure was predicted by knowing someone who did something that would make someone else repost (β = .16, *p* = .03) even with positive (β = .24, *p* < .01) and negative (β = –.28, *p* = .001) perceptions entering the model. Similarly, for harmfulness, closeness to someone who did something that might make someone else repost entered the model (β = –.19, *p* = .01) in addition to negative perceptions of the movement (β = –.29, *p* < .001).

A regression with an MLR was run for the blame variables due to skewness of these variables. Knowing someone who did something that would make someone else repost predicted the amount of blame allocated to Zoe (β = –.01, *p* = .04).

### How Do Participants Respond to a Potentially Threatening Video of #MeToo?

#### Emotional response

Of this sample, 123 participants watched the #MeToo video (48.6%) and 130 participants watched the environmental video (51.4%). The PANAS was completed by each participant, after watching the video, to rate how the video made them feel. We conducted independent sample *t* tests to assess whether men and women differed in their emotional response to either video. The results are displayed in Table 7

**Table 7.** Descriptive Statistics of PANAS-Scores for Men and Women for Both Videos and Independent Sample *t* tests.

PANAS	#MeToo					Environmental				
	Men		Women		*t*	Men		Women		*t*
	*M*	*SD*	*M*	*SD*		*M*	*SD*	*M*	*SD*	
Upset	2.82	1.30	3.11	1.10	−1.29	2.62	1.15	2.97	1.26	−1.62
Hostile	2.18	1.22	2.07	1.05	0.51	2.04	1.25	1.93	1.28	0.49
Alert	3.35	1.17	3.65	1.02	−1.50	3.75	.98	3.85	1.15	−0.53
Ashamed	2.19	1.32	1.65	1.00	2.56*	2.71	1.35	2.77	1.32	−0.27
Inspired	2.88	1.34	3.60	1.36	−2.93**	3.55	1.22	3.80	1.15	−1.21
Nervous	2.01	1.20	1.91	1.09	0.50	2.36	11.32	2.43	1.23	−0.32
Determined	2.84	1.30	3.33	1.29	−2.08*	3.53	1.28	3.59	1.06	−0.30
Attentive	3.56	1.10	3.85	1.15	−1.46	3.91	1.01	4.07	.96	−0.88
Afraid	1.94	1.27	1.65	.96	1.41	2.33	1.27	2.56	1.37	−0.97
Active	2.94	1.26	2.87	1.23	0.30	3.52	1.12	3.23	1.28	1.39

*Note*. PANAS = Positive and Negative Affect Schedule.

*p < .05. **p < .01. ***p < .001.

. For the #MeToo video, three significant differences emerged. Men recorded significantly more shame than women. Furthermore, women reported higher levels of feeling both inspired and determined. No significant differences emerged for the environmental video.

We performed two-way analyses of variance (ANOVAs) for each PANAS variable to determine whether there was an interaction effect of gender and video for any of the variables, but no significant interactions emerged.

#### Disclosure response

We examined the interaction effects of gender and video on the vignette variables. For belief in the vignette, a two-way ANOVA with gender and video as predictors and belief in vignette as the outcome variable was used. No significant interaction effect was found. Similar analyses were conducted for the harmful and avoidable variables. No significant interaction effects were found.

With respect to the blame variables, a multiple regression with dummy variables was conducted (with a robust maximum likelihood estimator for the blame allocated to Zoe and Zoe’s mom’s boyfriend). None of the interaction effects were significant.

## Discussion

The aim of this study was to examine exposure to and perceptions of the #MeToo movement, and to assess how these factors related to participants’ responses to sexual violence. Most participants had heard of the #MeToo movement, and 17% reported they had reposted the status. Most participants knew someone who had reposted the status and close to half knew someone who had done something to make someone else repost #MeToo.

These findings suggest that #MeToo is a well-known movement and most people have had first- or secondhand exposure to it. As hypothesized, a gender difference emerged with women more likely to know someone who had reposted the status. Interestingly, the results indicated that men were marginally more likely to be close to someone who had done something to cause someone else to repost, highlighting the importance of further exploring the concept of proximity to victimization as well as perpetration.

The observed gender differences are in line with previous research that suggests women tend to have a closer proximity to victimization through relationships with victims (Miller & Cromer, 2015). Although a gender difference in reposting the status was not found in this study, the number of participants who reposted was small. Furthermore, results suggested that people who reposted the status were more likely to know someone who had done something to make someone else repost. It may be that participants were thinking of the person who did something to them that made them repost the status. Given that most people are victimized by someone they know well (National Institute of Justice, 2003), that could explain this finding. Future studies should attempt to tease out the overlap between one’s own victimization and knowing a perpetrator.

The perceptions of men and women of the #MeToo movement varied in nearly every aspect. Men held significantly more negative views than women, and women reported significantly more positive feelings associated with the movement. This finding is in line with the study by Kunst et al. (2019) who found a similar gender difference in perceptions of the movement. The negative perceptions held by men may be indicative of an underlying defensive response. It would appear then, that although the #MeToo movement seems to have a positive effect on women, making them feel empowered and heard, the opposite is true for men. This finding highlights the polarizing nature of the movement and suggests that men may feel attacked by the movement. These feelings might make them less likely to engage in a conversation about sexual violence and to emerge as allies to women rather than as opponents. Indeed, Casey (2010) posed that defensiveness reduces the likelihood of people stepping up as allies. It may be the case that polarization, as a result of #MeToo, is leading to more skepticism, resulting in a less supportive response to a disclosure of sexual violence.

With regard to the responses participants gave to the CSA disclosure, the responses skewed toward positive, with most participants responding in a non-skeptical, supportive manner. However, several differences in responses did emerge, and they highlight how perceptions of a broader movement may shape responses in a specific situation. This finding is similar to findings from research into the Black Lives Matter movement, which suggest attitudes toward the movement are related to how specific situations are perceived (e.g., Reinka & Leach, 2017). Indeed, participants who reported having more positive views of #MeToo were also more likely to respond to a disclosure in a supportive manner. From the specific vignette questions, it appeared that when participants held more negative views of the movement, they were more likely to blame Zoe’s mom and to find the vignette more avoidable. Further research should investigate the nature of this seeming diffusion of blame. Furthermore, because we did not ask participants about their perceptions of sexual violence before the #MeToo movement, we cannot be certain that it is the movement itself that shaped their perceptions. A future study might ask participants explicitly whether their views on sexual violence have changed as a result of the #MeToo movement.

When looking at victimization proximity and perceptions, results showed that reposting the status was, on one hand, more likely to be associated with negative perceptions of the movement for female participants but, on the other hand, male participants were more likely to feel positive as well as negative emotions about the movement. These results may have emerged due to the small number of participants who reported having reposted the status. However, at least in part, these results may also suggest a difference in the individual experiences of the movement and their victimization. Kunst et al. (2019) also found that people who had a personal history of sexual victimization perceived the movement as less beneficial than those who did not.

Perhaps, #MeToo is viewed as not doing enough to address victimization or as not providing any tangible support for victims. Rape, Abuse, & Incest National Network’s (RAINN) guidelines on how to respond to victims of sexual violence emphasize the importance of continued support. A very public reposting without subsequent ongoing support may end up being more harmful than helpful to a victim (Ullman, 2002). Furthermore, participants who reposted the status may simply have stronger perceptions of the movement in general due to the personal relevance of the movement for them (e.g., Lang & Bradley, 2010). A further explanation may be drawn from an article on hashtag feminism and, specifically, a similar hashtag that trended on social media in 2014 (Mendes et al., 2018). The hashtag #BeenRapedNeverReported similarly sought to draw attention to the prevalence of sexual violence and to highlight the problem of underreporting. The researchers interviewed people who had posted the hashtag on social media and they found a similar tension between positive and negative emotions, with participants reporting finding the experience comforting on one hand, but also triggering on the other hand. Similarly, the interviews showed that the public nature of a hashtag leads to support from outside one’s own social circle, but for 72% of participants, it also led to hostility, threats, and misogynistic online abuse. The present findings of mixed emotions may be the result of similar conflicting processes. Finally, having reposted the status provides no further information of the motivations for doing so. Instead, it means the person identifies as a victim of some form of sexual violence and/or harassment. As such, the range of victimization experiences is likely to vary, and victims of different types of experiences may experience the #MeToo movement differently. Reposting the status suggests that the person is an “acknowledged” victim (Koss, 1985), meaning they identify as a victim.

However, the experience of unacknowledged victims, who do not view their experience as a victimization, remains hidden although they experience similar negative consequences as acknowledged victims (Frazier & Seales, 1997; Kahn & Mathie, 2000; Orlando & Koss, 1983). In short, the heterogeneous nature of victimization likely plays a role in the range of emotional responses reported in this study.

Knowing someone who reposted the status was linked with more positive perceptions of #MeToo, and closeness to someone who did something to cause someone else to repost the status was linked with more negative perceptions. It is important to point out that the phrasing of this question may have led some people to confuse posting something about #MeToo, with reposting the status, which is synonymous with a public disclosure of sexual victimization. Interestingly, proximity to victimization did not have an effect on the vignette variables, whereas proximity to perpetration did.

This may be due to the nature of #MeToo, and closeness to someone who reposted may not be strong enough to create differences in responses. #MeToo is very public by nature, as evidenced by the public reposting of the status on social media with the intent of giving visibility to sexual victimization. However, due to the public nature of reposting the status, it is likely not a great measure of proximity to victimization because no further conversation with the person who reposted may have occurred. A better indicator of proximity to victimization would be to ask whether the participant knows anyone who has been a victim of sexual violence or harassment.

Interestingly, participants who knew someone who had done something to make someone else repost #MeToo were more likely to believe the vignette, but closeness to this person was not a factor. This finding suggests there may be an availability bias at play, with people who know someone who caused someone else to repost the status being more likely to recall an incident of sexual violence, which in turn could increase belief in the vignette. Such a bias may indeed occur following media exposure to a phenomenon (e.g., Iyengar, 1990). On the contrary, closeness to such a person was linked to viewing the vignette as less harmful and to allocating more blame to the victim. The dissonance between belief that the disclosure is real and simultaneous minimization of harm seem indicative of a type of defensiveness, or at least cognitive dissonance, whereby the participant tries to resolve the conflict of believing in victimization while also knowing someone who victimized someone else.

Interestingly, gender differences were found for the #MeToo video in how it made participants feel, with men reporting more shame and women reporting being inspired and determined. These differences suggest that, to an extent, the video did have the desired effect of eliciting different, if not opposite, responses in men and women. Future studies may wish to further examine the nature of this shame reported by men. For example, shame is defined as a painful feeling with negative effects on interpersonal relations. However, shame and guilt are often confused, with guilt having comparatively more adaptive behaviors such as taking responsibility for one’s actions (Tangney & Dearing, 2003). It is likely that these emotions would have a different effect on how people respond to movements such as #MeToo.

This study had some limitations. First, at no point were participants asked whether they had ever done something that would cause someone else to repost #MeToo. If so, this would be the closest possible proximity to perpetration and that would likely skew the results. Similarly, participants who reposted #MeToo were also asked whether they knew someone who did something that caused someone else to repost the status, which means they could have thought of the person who caused them to repost. This is clearly a different kind of proximity to perpetration than the target of this study, and thus, this should be considered in future research. Second, we presented participants with response options regarding their perceptions of the movement and, in doing so, we may have superimposed bias onto them. A future study may ask participants to write in their views on the movement instead to allow for a more objective reflection of their views. Third, the #MeToo movement is very public in nature. Reposting a status for other people to see means that proximity to victimization increases for everyone who uses social media as it takes away the intimate nature of such disclosure in a more personal manner. As such, knowing someone who reposted the status may not be the best indication of proximity to victimization. Furthermore, although use of Amazon’s Mechanical Turk may result in a more diverse sample than an undergraduate student sample, MTurk samples are also not representative of the U.S. population (Arditte et al., 2016). Finally, we did not explicitly ask people whether the #MeToo movement had changed their perceptions of sexual violence and harassment, and thus we cannot conclude whether the movement directly influenced participants’ responses to the vignette.

This study confirmed that the #MeToo movement is well-known, and that most people have heard of it. We studied well-established gender differences in response to sexual violence in the broader context of the #MeToo movement. It appears that women are more likely to have positive perceptions of the movement than men, and these perceptions are reflected in responses to a CSA disclosure. Future research should explore whether men experience greater fear of being accused of sexual violence in light of increased disclosures as a result of the movement. Self-identified victims of sexual violence may experience the movement in different ways. Beyond the scope of #MeToo, this article sheds light on the challenges victims of sexual violence may face when disclosing. Closeness to a victim increases the likelihood for positive responses, whereas closeness to a perpetrator has the opposite effect. This finding makes a case for the importance of social relationships in creating a safe environment for victims to disclose. Although belief in a disclosure seemed unaffected, the impact on blame allocation and perceived harmfulness suggests a secondary, defensive response to disclosure that should be further explored.

## Supplemental Material

sj-pdf-1-jiv-10.1177_0886260520925781 - Supplemental material for Empowerment or Threat: Perceptions of Childhood Sexual Abuse in the #MeToo Era Click here for additional data file. Supplemental material, sj-pdf-1-jiv-10.1177_0886260520925781 for Empowerment or Threat: Perceptions of Childhood Sexual Abuse in the #MeToo Era by Melissa S. de Roos and Daniel N. Jones in Journal of Interpersonal Violence
