# Supplementary material for: Empowerment or Threat: Perceptions of Childhood Sexual Abuse in the #MeToo Era
Source: J Interpers Violence. 2020 Jun 6;37(7-8):NP4212–37. doi: 10.1177/0886260520925781 (PMC8980449; doi:10.1177/0886260520925781)
Supplement: sj-pdf-1-jiv-10.1177_0886260520925781 - Supplemental material for Empowerment or Threat: Perceptions of Childhood Sexual Abuse in the #MeToo Era [file sj-pdf-1-jiv-10.1177_0886260520925781.pdf]

Supplementary Table 1

Sample Characteristics split by Gender

	Male (%)	Female (%)
**Age**	*M*=35.73; *SD*=11.86	*M*=35.09; *SD*=11.86
Range	18-75	19-66
**Sexual Orientation**		
Straight	122 (89.1%)	102 (87.9%)
Gay or lesbian	3 (2.2%)	0 (0.0%)
Bisexual	11 (8.0%)	13 (11.2%)
Prefer not to say	16 (11.7%)	1 (0.7%)
**Education**		
Bachelor's degree	63 (46.0%)	47 (40.5%)
Some college, no degree	28 (20.4%)	19 (16.4%)
Graduate degree	17 (12.4%)	24 (20.7%)
Associate degree	17 (12.4%)	13 (11.2%)
High school degree or equivalent	11 (8.0%)	10 (8.6%)
Less than high school degree	1 (0.7%)	2 (1.7%)
Missing data	0 (0.0%)	1 (0.9%)
**Race/Ethnicity**		
White (non-Hispanic)	92 (67.2%)	87 (75.0%)
Black or African American	10 (7.3%)	11 (9.5%)
Asian	15 (10.9%)	6 (5.2%)
Hispanic	10 (7.3%)	7 (6.0%)
Multiple races	8 (5.8%)	0 (0.0%)
American Indian/Alaskan Native	1 (0.7%)	2 (1.7%)
Other race	1 (0.7%)	2 (1.7%)
Missing data	0 (0.0%)	1 (0.9%)
**Employment**		
Employed – working >40hrs per week	103 (75.2%)	68 (58.6%)
Employed – working 1-39 hours per week	18 (13.1%)	31 (26.7%)
Not employed – looking for work	8 (5.8%)	5 (4.3%)
Not employed – not looking for work	0 (0.0%)	11 (9.5%)
Retired	7 (5.1%)	1 (0.9%)
Disabled – not able to work	1 (0.7%)	0 (0.0%)
**Parent**		
Yes	70 (51.1%)	66 (56.9%)
No	50 (36.5%)	45 (38.8%)
Missing data	17 (12.4%)	5 (4.3%)
